# Unusual Presentation of Meckel's Diverticulum: Gangrene due to Axial Torsion

**DOI:** 10.1155/2015/571847

**Published:** 2015-02-22

**Authors:** Ahmet Rencuzogullari, Kubilay Dalci, Orcun Yalav

**Affiliations:** Department of General Surgery, Cukurova University Medical Faculty, 01330 Adana, Turkey

## Abstract

Meckel's diverticulum is the most common congenital anomaly of the small bowel. The majority of cases are asymptomatic; however, life-threatening complications can also take place. We present a case of a 37-year-old male who was admitted with symptoms of acute, severe abdominal pain in the right iliac fossa. The patient was operated on with the preoperative diagnosis of acute appendicitis but the operative findings were consistent with torted Meckel's diverticulum due to presence of mesodiverticular band and he was treated successfully with surgical resection.

## 1. Introduction

Meckel's diverticulum (MD) is the most common congenital anomaly of the small bowel, which was first described by Johann Friedrich Meckel in 1809. MD is a true diverticulum that develops from the obliteration of the omphalomesenteric duct during embryonic development. The incidence of this entity, in autopsy and retrospective studies, ranges from 0.14 to 4.5 percent [1]. Most commonly, MD remains asymptomatic. Complications of MD include hemorrhage associated with peptic ulceration, due to heterotopic gastric mucosa located within the diverticulum and intestinal obstruction due to banding, volvulus, intussusception, tumor formation, and axial twisting around its base [2, 3]. Preoperative diagnosis of complicated MD is challenging because the symptoms can mimic a variety of more common ailments such as appendicitis. We hereby report a case with torted Meckel's diverticulum due to presence of mesodiverticular band.

## 2. Case Report

**Figure 1:**
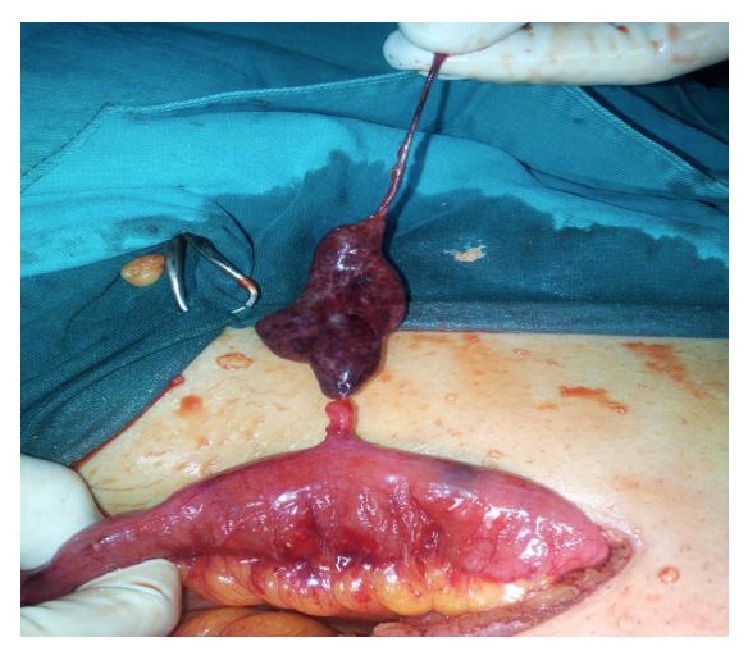
Torsion of Meckel's diverticulum around the base and necrosis.

**Figure 2:**
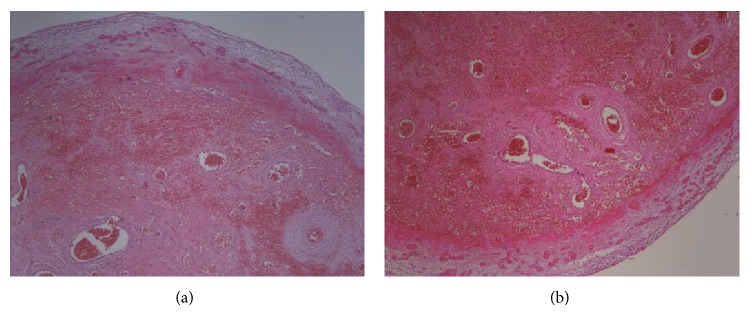
(a) Transmural necrosis and hemorrhage (H&E staining ×100 magnification). (b) Ischemic necrosis, congestion, and hemorrhage (H&E staining ×100 magnification).

A 37-year-old man presented to our hospital with an 8-hour history of acute, severe, and right lower abdominal pain. The physical examination of patient revealed defense and guarding in the right lower quadrant, with rebound tenderness and altered bowel sounds. The leukocyte count was elevated at 13.7 × 10^9^/L, and a differential count demonstrated 90% segmented neutrophils. An abdominal X-ray showed minimally dilated small bowel loops. The patient was operated on with the preoperative diagnosis of acute appendicitis. The appendix, however, intraoperatively was normal; thus, the incision was extended. The torted MD was observed 60 cm proximal to the ileocecal valve and had distal necrosis (Figure 1). The rest of the bowel appeared normal. MD was resected by using a stapling device. Pathological examination confirmed the diagnosis of MD with gangrene (Figures 2(a) and 2(b)). The recovery period was uncomplicated and the patient was discharged on postoperative day 5.

## 3. Discussion

This case report describes an unusual presentation of torted MD with gangrene in a patient who was operated on with a preoperative diagnosis of acute appendicitis. Malhotra et al. in their case report published in 1998 mention only 4 adult cases and 1 pediatric case with axial torsion of MD. However, a literature review that we conducted has shown that since then an additional 19 cases have been reported [4].

MD is a true diverticulum that develops from the vestige of the omphalomesenteric duct during embryonic development and is reported to be infrequent [2]. Clinically the majority of MD is asymptomatic and is called silent MD; however, life-threatening complications can also take place. The lifetime risk of patients with MD to develop complications was shown in a study to be 6.4% [5]. The most common of these complications is lower gastrointestinal system (GIS) bleeding, resulting from ulceration caused by ectopic gastric mucosa. Other major complications include obstruction, intussusception, diverticulitis, perforation, and development of malignancies. However, preoperatively diagnosing MD is often difficult, with only 6–12% of cases being diagnosed correctly [6]. Most commonly, the symptoms of a complicated MD simulate acute abdomen due to appendicitis; thus, appendicitis is usually the main preoperative diagnosis, while differential diagnoses include small bowel obstruction, acute cholecystitis, and liver abscess [4, 7–9]. In our case, similarly the preoperative diagnosis was acute appendicitis. Previous reports have put forth that despite improvements in radiodiagnostics, only 4% of such cases are accurately diagnosed [1]. As mentioned before, axial torsion is already the rarest complication and development of gangrene in this clinical context is even rarer. Our literature review has identified only 9 [4, 6, 7].

The mechanism of axial torsion of MD has not yet been described successfully. One explanation could be the MD having a wide body but narrow base and the presence of mesodiverticular band [4]. The macroscopic appearance of our case's MD supports this conjecture. Other possible risk factors for torsion have also been reported in the literature. A neoplasm arising within MD is one of the rare presentations mentioned despite being exceptionally rare [8].

The treatment of symptomatic MD is surgical resection. Wedge resection usually suffices; however, under some circumstances, segmental ileum resection with end-to-end anastomosis may be indicated. Resection for incidental asymptomatic MD cases is questionable. However, in any case, resection of fibrous bands is recommended to prevent further complications such as torsion and obstruction [6]. Increasing number of reports has begun recommending use of laparoscopy for treatment [10].

## 4. Conclusion

Meckel's diverticulum complications should be considered in the differential diagnosis of an acute appendicitis case, especially when intraoperatively a normal appendix is identified. Imaging has not been shown to be effective in identifying a torted MD; therefore, surgical vigilance, suspicion, and consideration are vital to recognize.
